# Association between obesity and anemia in an nationally representative sample of United States adults: a cross-sectional study

**DOI:** 10.3389/fnut.2024.1304127

**Published:** 2024-03-13

**Authors:** Zhuo Chen, Bingyan Cao, Lu Liu, Xudong Tang, Hao Xu

**Affiliations:** ^1^Xiyuan Hospital, China Academy of Chinese Medical Sciences, Beijing, China; ^2^Jining Hospital of Traditional Chinese Medicine, Jining, China; ^3^Cardiovascular Diseases Center, Xiyuan Hospital, Beijing, China

**Keywords:** anemia, obesity, body fat percentage, iron deficiency, inflammation, NHANES

## Abstract

**Introduction:**

Few studies are about the relationship between anemia and obesity, and previous studies have only paid attention to BMI.

**Methods and Results:**

We first included body fat percentage (BF%) as an assessment indicator and divided it into quartiles, grouped participants into obesity and non-obesity used data from NHANES database. After adjustment for age, gender, ethnicity, education and family income, the level of soluble transferrin receptor (sTfR), and incidence of elevated CRP or HsCRP were progressively higher with increased BF%, whereas mean cell volume (MCV), natural logarithm (Ln) serum ferritin (SF), and Ln SF/sTfR were progressively reduced. Although a higher prevalence of anemia and lower hemoglobin was observed with increased BF%, but there was no statistical difference. Women in the highest BF% group demonstrated a significantly higher risk of iron deficiency compared to those in the lowest BF% group.

**Discussion:**

BF% should be given more attention, and women with high BF% should pay attention to iron deficiency.

## Introduction

The global prevalence of obesity has been accelerated by increases in national income, coupled with a lack of physical activity and nutritionally balanced diets. Over the past 40 years, the obesity rate has risen from 3 to 11% in men and from 6 to 15% in women (1). By 2030, it is foreseen that almost half of the adult population in the United States will be obese (2).

Obesity is associated with multiple adverse outcomes, including anemia, which is a serious global public health problem. In addition to height and weight, anemia is a basic indicator that reflects the nutritional well-being of individuals. While it may appear paradoxical, obesity is also linked to nutrient deficiencies (3), most people with long-term anemia looks thinner than others. The overlooked paradox of the coexistence of obesity and anemia certainly exist. An assessment tool that has been widely used in clinical practice and research as a screening tool for obesity is the BMI. However, BMI cannot differentiate body composition or excess fat distribution. This measurement alone is insufficient to evaluate adiposity-related disease risk.

Anemia’s clinical manifestations often include fatigue, pallor, shortness of breath, and an increased heart rate. Iron deficiency anemia (IDA) stands as the prevailing cause of anemia worldwide, while anemia of inflammation (AI), also known as anemia of chronic disease (ACD), is recognized as the most frequent form of anemia in hospitalized and chronically ill patients, which ranks as the second most common type of anemia globally, following IDA (4). Obesity is characterized by low-grade chronic inflammation. This results in the production of certain inflammatory cytokines, leading to elevated levels of circulating plasma inflammatory markers and inflammatory cells (5). Body mass index (BMI)-based studies have shown that obese women are associated with iron deficiency (6). Body fat percentage (BF%) is a better indicator for classifying obesity compared to BMI (7), while little attention has been paid to the relationship between BF% and anemia, iron, and inflammation.

## Methods

### Study design and population

National Health and Nutrition Examination Survey (NHANES) utilized a stratified multistage probability sample to represent the civilian, non-institutionalized population of the United States. The National Center for Health Statistics (NCHS) of the Centers for Disease Control and Prevention conducted surveys through household interviews, followed by standardized physical examinations at Mobile Examination Centers (MECs). The NHANES protocol has been approved by the NCHS Research Ethics Review Board. NHANES data are released on a 2-year cycle. The data collection and analysis procedures for NHANES have been published (available from https://www.cdc.gov/nchs/nhanes/index.htm).

**Figure 1:**
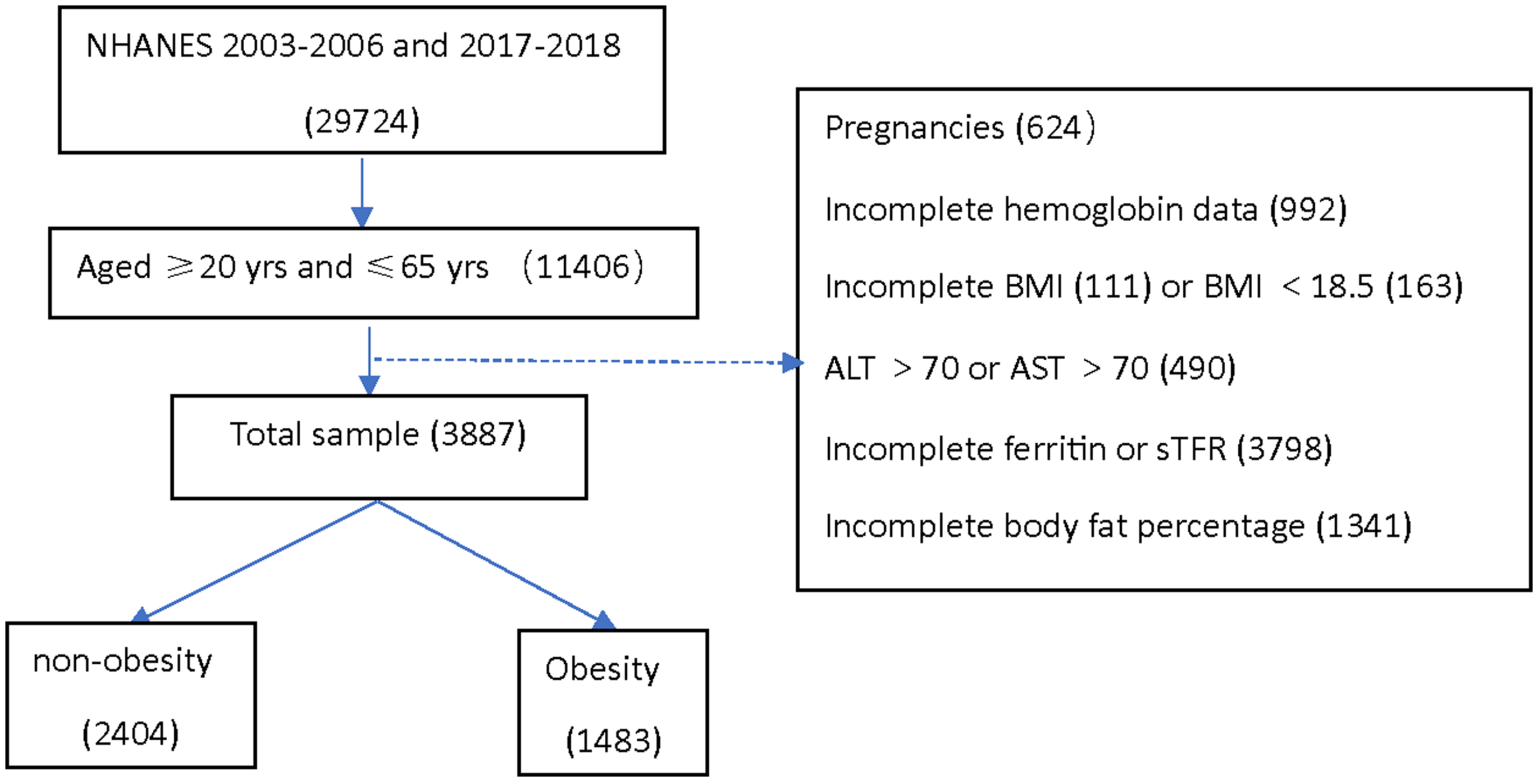
Cohort selection.

This analysis was conducted using three data cycles of NHANES: 2003–2004, 2005–2006, and 2017–2018. Institutional review board approval was not required for the current analysis. The analytical sample included adults aged 20–65 years who were not pregnant at the time of participation and had no missing data for hemoglobin, serum ferritin (SF), soluble transferrin receptor (sTfR), BMI, and Dual-Energy X-ray Absorptiometry (DEXA)-whole body measurements. Individuals who potentially had liver disease were excluded from the study, with abnormal liver enzyme levels defined as alanine aminotransferase (ALT) > 70 U/L or aspartate aminotransferase (AST) > 70 U/L (8) (Figure 1).

### Laboratory methods

#### Serum ferritin

Serum ferritin was measured by electrochemiluminescence immunoassay. Over the years, various laboratory measurement methods have been employed for sample analysis. Due to variations in the testing methods, the NHANES team conducted cross-studies to compare the data and made necessary adjustments based on the comparative results before releasing the data to the public. For ferritin, two methods were used in 2003–2004. The National Center for Environmental Health analyzed all 2003 samples with a BioRad assay (BioRad Laboratories, Hercules, CA, United States) and all 2004 samples with a Roche/Hitachi assay (Roche Diagnostics, Basel, Switzerland). NHANES used three piecewise linear regression equations to adjust the 2003 ferritin data to be comparable to the 2004 ferritin (ng/mL) data before publishing the data. Ferritin in 2005–2006 is measured using immuno-turbidimetry method on Roche/Hitachi 912 clinical analyzer. Ferritin in 2017–2018 was measured using sandwich principle on Roche Cobas® e601. We converted 2017–2018 data from E170 to Hitachi 912, for ferritin in 2017–2018 to be accurately comparable to other years, a Deming regression analysis was performed, and the following regression was obtained for Ferritin (ng/mL):

$$E170\;\left(2017:2018\right)=10^{**}\left(0\cdot 989^{*}\log\;10\;\left(\mathrm{Hitachi}\;912\right)+0\cdot 049\right)$$

#### Soluble transferrin receptor

Soluble transferrin receptor is measured using immuno-turbidimetry Roche kits on the Hitachi 912 clinical analyzer in 2003–2004 and 2005–2006, on the Cobas® c501 clinical analyzer in 2017–2018. There was no need for adjustment of the sTfR measured value. The ratio of natural logarithm (Ln) SF/sTfR reflected iron levels in the body (9). “Low” levels of Ln SF/sTfR were defined as values below the 25th percentile (< 0.818 for women, < 1.412 for men), seen as iron deficiency (ID).

#### Complete blood count

Hemoglobin, mean cell volume (MCV) and mean corpuscular hemoglobin concentration (MCHC) are derived from complete blood count (CBC) with five-part differential—whole blood. The methods used to derive CBC parameters are based on the Beckman Coulter (DxH 800 instrument) methodology of counting and sizing, in combination with an automatic diluting and mixing device for sample processing, and a single beam photometer for hemoglobinometry. Anemia is defined as hemoglobin level less than 13 g/dL for men and less than 12 g/dL for women (10).

#### C-Reactive protein

National Health and Nutrition Examination Survey 2003–2004 and 2005–2006 quantified C-reactive protein (CRP) by latex-enhanced nephelometry in University of Washington, Seattle, WA, United States. Analysis of high sensitivity C-reactive protein (HsCRP) in NHANES 2017–2018 is by a two-reagent, immunoturbidimetric system, using the Roche Cobas 6000 chemistry analyzer (Cobas 6000) in University of Minnesota—Advanced Research Diagnostics Laboratory (ARDL), Minneapolis, MN, United States. Elevated CRP was defined as CRP more than 8 mg/L. Elevated HsCRP was defined as HsCRP more than 2 mg/L (11).

### Anthropometry

The BMI is derived from height and weight measurements collected at the Mobile Examination Centers (MECs). Obesity was assessed using BMI and classified as non-obese (BMI < 30) and obese (BMI ≥ 30) (12). Total BF% measurements were performed using whole-body dual-energy X-ray absorptiometry (DXA) scanning (Hologic, Inc., Bedford, Massachusetts).

### Assessment of potential confounding variables

Gender, race/ethnicity, age, education, marriage, and income status, which may be associated with BF% were included as potential confounders in the regression models. Race/ethnicity was categorized into five groups: non-Hispanic white, non-Hispanic black, Mexican American, other Hispanic, and other race. Education was categorized as “no college degree” and “college degree.” Marriage was categorized into living with a partner or not. Income status was categorized as having an annual income of more than $20,000 or less than $20,000. Current smoking was defined as smoked at least 100 cigarettes in life and smoked within the last 30 days. Alcohol consumption was defined as having ≥3 alcohol drinks per year. Blood donations were defined as participants had donated blood in the past 12 months.

### Statistical analyses

Statistical analysis was performed using IBM SPSS Statistics software (version 28, IBM Corp., Armonk, NY, United States), which incorporated weights to account for the complex sample design. The 6-year examination weights from the NHANES data for 2003–2006 and 2017–2018 were utilized to account for non-response and oversampling in all analyses. We assessed whether there were differences in characteristics between non-obesity and obesity in the population eligible for inclusion using χ^2^ or *t* tests (*p* < 0.05; two-tailed). We log-transformed SF (Ln SF) data to standardize the distribution. BF% was put into quartiles. After adjusting for the potential confounders such as age, race, education, family income, and logistic regression models compared differences in hemoglobin concentration (HB), MCV, SF, and sTfR levels between populations with different BF%. The characteristics of anemia and non-anemia in obese people were compared according to men and women separately using χ^2^ or *t* tests. We performed further analysis on men and women to look for any sex-specific differences in the association between BF% and SF, and sTfR, HB, and CRP.

## Results

Of the 3,887 NHANES participants analyzed in the current study, 1,483 were obesity, 2,404 were non-obesity. The mean ± SE for age was 37.64 ± 0.27 years. The proportion of men was 24.5%. Obesity was associated with higher age, a higher prevalence of non-Hispanic Black, no college education, Income poverty, elevated SF and sTfR, reduced MCV and MCHC, elevated prevalence of anemia and increased incidence of elevated CRP or HsCRP (Table 1). Blood donation in the last 12 months did not differ statistically by BMI category. No interaction between blood donation and anemia (*p* = 0.92, Pearson test).

**Table 1:** Characteristics of participants presented by BMI category.

	Overall	Non-Obesity	Obesity	*p* value
		BMI 18.5–29.9 kg/m^2^ (*n* = 2,404)	BMI ≥ 30 kg/m^2^ (*n* = 1,483)	
Men, *n* (%)	943 (24.5)	593 (23.4)	350 (26.4)	0.248
Age, year, mean (SE)	37.64 ± 0.27	36.91 ± 0.41	38.91 ± 0.31	<0.001
Race, *n* (%)				<0.001
Mexican American	729 (10.2)	421 (9.1)	308 (12.2)	
Other Hispanic	281 (6.6)	173 (6.6)	108 (6.7)	
Non-Hispanic White	1,459 (62.5)	946 (64.3)	513 (59.3)	
Non-Hispanic Black	847 (11.7)	419 (9.2)	428 (16)	
Other Race—including multi-racial	571 (9)	445 (10.8)	126 (5.8)	
Married/living with a partner, *n* (%)	2,335 (62.1)	1,489 (63.1)	846 (60.4)	0.345
Ever attended college, *n* (%)	2,298 (64.8)	1,477 (67.9)	821 (59.5)	<0.001
Annual income > $20,000, *n* (%)	730 (13.4)	413 (11.8)	317 (16.1)	0.003
Current smoking, *n* (%)	717 (20.9)	442 (21.1)	275 (20.4)	0.673
Alcohol consumption, *n* (%)	841 (35.8)	511 (34.9)	330 (37.5)	0.325
Donated blood in past 12 months, *n* (%)	200 (6.3)	117 (5.9)	83 (6.8)	0.202
Ln SF	4.05 ± 0.03	3.99 ± 0.03	4.16 ± 0.04	0.005
sTfR (mg/L)	3.40 ± 0.04	3.24 ± 0.04	3.68 ± 0.07	<0.001
Hb (g/dL)	13.95 ± 0.04	13.97 ± 0.04	13.93 ± 0.07	0.601
MCV (fl)	88.77 ± 0.17	89.71 ± 0.17	87.13 ± 0.23	<0.001
MCHC (g/dL)	30.02 ± 0.06	30.39 ± 0.06	29.37 ± 0.09	<0.001
Anemia, *n* (%)	308 (5.7)	157 (4.9)	151 (7)	0.02
Elevated CRP or HsCRP, *n* (%)	1,272(31)	463 (18.3)	809 (53.3)	<0.001

Hb, Hemoglobin; MCV, Mean cell volume; Ln SF, Ln serum ferritin; sTfR, Soluble transferrin receptor; MCHC, Mean corpuscular hemoglobin concentration; CRP, C-Reactive protein; and Hs-CRP, Hypersensitive C-reactive protein.

After adjustment for age, gender, ethnicity, education and family income, the level of sTfR and percentage of elevated CRP or HsCRP were progressively higher with increased BF% category, whereas MCV, Ln SF, and Ln SF/sTfR were progressively reduced (Table 2). Although as shown in the Table 2, a higher prevalence of anemia and lower hemoglobin was observed with increased BF%, but they did not have statistically significant after adjustment for the confounders.

**Table 2:** Comparison of anemia, Hb, MCV, Ln SF, Ln SF/sTfR, and elevated CRP or HsCRP of all participants by BF% quartiles.

	1st quartile BF%	2nd quartile BF%	3rd quartile BF%	4th quartile BF%	Unadjusted *p* value	Adjusted *p* value
Anemia	40 (2.9)	59 (4.3)	94 (6.9)	115 (9)	<0.001	0.633
Hb (g/dL)	14.54 ± 0.05	14.01 ± 0.06	13.66 ± 0.05	13.51 ± 0.06	<0.001	0.400
MCV (fl)	89.62 ± 0.24	89.44 ± 0.25	88.70 ± 0.23	87.12 ± 0.22	<0.001	<0.001
sTfR (mg/L)	3.00 ± 0.06	3.25 ± 0.06	3.55 ± 0.07	3.87 ± 0.07	<0.001	<0.001
Ln SF	4.47 ± 0.05	4.02 ± 0.05	3.81 ± 0.04	3.85 ± 0.33	<0.001	0.001
Ln SF/sTfR	1.67 ± 0.38	1.43 ± 0.03	1.28 ± 0.02	1.18 ± 0.02	<0.001	<0.001
Elevated CRP or HsCRP, *n* (%)	223 (22.2)	237 (22.4)	299 (29.7)	513 (52.2)	<0.001	<0.001

Hb, Hemoglobin; MCV, Mean cell volume; Ln SF, Ln serum ferritin; sTfR, Soluble transferrin receptor; CRP, C-Reactive protein; and Hs-CRP, Hypersensitive C-reactive protein.

Adjusted for: age, race, education, and family income.

Further analysis targeting only obese groups showed that anemic women had lower levels of SF and higher levels of sTfR compared to non-anemic women. On the other hand, among anemic men, the percentage of individuals with elevated CRP or HsCRP levels was higher compared to non-anemic men (Table 3).

**Table 3:** Comparison of non-anemia and anemia in the obese population by sex.

	Women		Adjusted *p* value	Men		Adjusted *p* value
	Non-anemia	Anemia		Non-anemia	Anemia	
sTfR (mg/L)	3.58 ± 0.05	7.28 ± 0.44	<0.001^*^	3.01 ± 0.07	3.11 ± 0.41	0.569
Ln SF	3.97 ± 0.04	2.44 ± 0.11	<0.001^*^	5.07 ± 0.07	5.59 ± 0.64	0.458
Elevated CRP or HsCRP, *n* (%)	516 (51.9)	76 (49.5)	0.545	208 (57.3)	9 (100)	0.034^*^

^*^p<0.05. Adjusted for: age, race, education, and family income.

sTfR, Soluble transferrin receptor; Ln SF, Ln serum ferritin; CRP, C-Reactive protein; Hs-CRP, Hypersensitive C-reactive protein.

**Figure 2:**
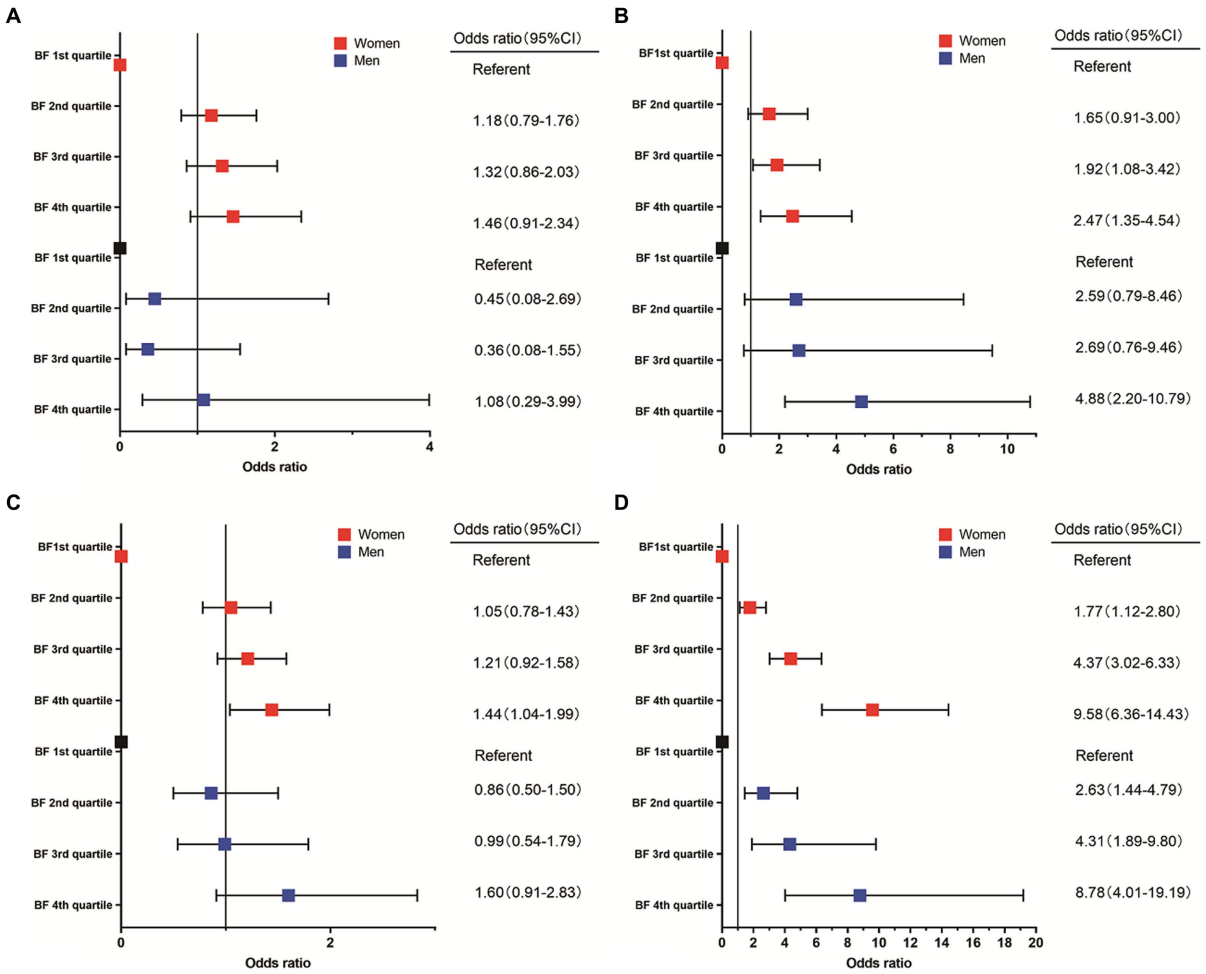
Association between incidence of anemia **(A)**, MCV ≤ 80 **(B)**, Ln SF/sTfR < the 25th percentile **(C)**, elevated CRP or HsCRP **(D)**, and body fat percentage.

We conducted subgroup analysis according to different genders. Similar results were observed in these subgroups in the adjusted model (Table 4; Figure 2). For women, compared with participants with lowest BF%, participants with highest BF% exhibited significantly increased risk of higher Ln SF/sTfR (OR, 1.44, 95% CI, 1.04–1.99) in multivariable analysis controlled for factors like age, race, education, and family income. Results of multivariable analysis showed a graded relation between MCV and obesity, with progressively higher odds of reduced MCV. For men, it showed 4th quartile BF% has the higher risk to be lower MCV compared to 1st quartile BF%. A graded relation between elevated CRP percentage and obesity was observed in both women and men. The higher the BF% is, the more likely they have an elevated CRP (C-reactive protein) ratio. There looked like a linear relationship observed between anemia, hemoglobin, and BF%; however, this relationship did not reach statistical significance.

**Table 4:** Association between total BF% and anemia, iron, inflammation by BF% quartiles by different gender.

Women	1st quartile BF% (lowest–34.8%)	2nd quartile BF% (34.8–39.7%)	3rd quartile BF% (39.7–43.9%)	4th quartile BF% (43.9–highest)
Anemia, *n* (%)	54 (5.2)	69 (6.6)	71 (8.1)	94 (9)
Hb (g/dL)	13.56 ± 0.05	13.57 ± 0.05	13.55 ± 0.06	13.52 ± 0.07
MCV (fl)	90.74 ± 0.22	89.19 ± 0.27	88.11 ± 0.26	87.02 ± 0.22
MCV ≤ 80	38(3.6)	69(6.2)	75(7.7)	102(9.7)
Ln SF/sTfR	1.28 ± 0.02	1.27 ± 0.02	1.26 ± 0.03	1.14 ± 0.02
Ln SF/sTfR (<0.818)	160 (19.2)	170 (19.8)	187 (22.4)	219 (25.6)
CRP or HsCRP, *n* (%)	79 (10.3)	137 (17.9)	270 (36.2)	408 (54.7)
Men	1st quartile BF% (lowest–23.8%)	2nd quartile BF% (23.8–27.9%)	3rd quartile BF% (27.9–31.7%)	4th quartile BF% (31.7–highest)
Anemia, *n* (%)	5 (1.9)	2 (0.8)	3 (0.7)	10 (1.9)
Hb (g/dL)	15.19 ± 0.06	15.07 ± 0.06	15.27 ± 0.10	15.27 ± 0.12
MCV (fl)	90.07 ± 0.52	87.94 ± 0.31	88.01 ± 0.48	88.02 ± 0.60
MCV ≤ 80	7 (1.4)	11 (2.6)	12 (2.7)	16 (4.7)
LnSF/sTfR	1.86 ± 0.06	1.93 ± 0.04	1.90 ± 0.07	1.85 ± 0.07
LnSF/sTfR(<1.412)	57 (21.8)	54 (18.4)	56 (19.6)	68 (28.9)
CRP or HsCRP, *n* (%)	41 (16.3)	84 (32.7)	98 (44.7)	155 (61.5)

Low level of Ln SF/sTfR defined as a value below the 25th percentile, <0.818 for women, <1.412 for men.

Hb, Hemoglobin; MCV, Mean cell volume; Ln SF, Ln Serum ferritin; sTfR, Soluble transferrin receptor; CRP, C-Reactive protein; and Hs-CRP, Hypersensitive C-reactive protein.

## Discussion

As far as we know, we are the first to use BF% instead of BMI to explore the link between obesity and iron deficiency and anemia. In the present large population-based cross-sectional representative sample of United States adults, we hypothesized that obesity may be associated with the features of IDA or AI. However, the outcome failed to demonstrate association between BF% and anemia.

Our results suggested that increasing BF% was associated with an increase in sTfR and incidence of elevated CRP or HsCRP; while on the other hand, with increasing BF%, there is an observed decrease in level of MCV, Ln ferritin and Ln ferritin/transferrin receptor. However, contrary to our hypothesis, although a decrease in HB with increasing BF%, there was no statistically significant correlation between changes in HB and the increased incidence of anemia. The prevalence of inflammatory conditions, as indicated by elevated levels CRP or HsCRP tended to increase in both women and men as BF% rose. There is no consensus on cutoffs on BF% in men and women separately, so we used the quartile method. ID was statistically significant in women compared 4th to 1st BF% group. There was a graded association between percentage of MCV ≤80 and BF% among women, which appears to be more robust. There was no statistically significant relationship between BF% and ID in men.

Lack of standards in diagnosis of ID posed challenges in accurately identifying ID (13, 14), some studies have used SF alone, as WHO has concluded that SF is a good marker of iron stores and should be used to diagnose ID in apparently healthy individuals (15). Some studies used serum iron and transferrin saturation, while few studies used sTfR. SF is not a sufficiently sensitive indicator for the diagnosis of ID in obese patients. The use of SF as the only biomarker for assessing ID may underestimate ID because obesity-associated chronic inflammation leads to elevated SF and SF may be an inflammatory marker rather than a marker of iron status in overweight and obese individuals (16). STfR is a promising candidate for the detection of ID. That is why our study also incorporated the use of sTfR, which is an inflammation-independent marker. This allows for the diagnosis of iron deficiency in patients with concurrent inflammation.

Various hypotheses have been proposed for the association between obesity and ID. One theory suggested that ID in obese subjects was due to nutritional imbalances (17). In some viewpoints, increased blood volume in obese individuals led to increased iron requirements (18). It was argued that the reason was decrease in iron-binding myoglobin in muscle due to decreased physical activity (19). While others believed that it was related to genetic predisposition (20). There were those who proposed that increased obesity was associated with decreased duodenal iron absorption (21, 22). Other physiological factors associated with chronic inflammation due to excessive obesity could also influenced the bioavailability of iron. The increased accumulation of total and visceral fat mass triggered the production of inflammatory cytokines (23). Chronic inflammation led to increased levels of feromodulin, a small peptide hormone that negatively regulated intestinal iron absorption. This hormone was inversely correlated with serum iron levels (24, 25). In addition, increased adipose tissue volume in obese individuals might directly contribute to increased ferredoxin expression (26). However, there was no difference in iron intake through diet between obese and non-obese individuals (27, 28).

Our findings were in line with some previous studies. For example, a meta-analysis reported that obese/overweight participants were more likely to develop ID compared to normal weight participants (29). There was also another review that concluded the opposite, and although ID appeared to be a typical manifestation of severe obesity, the review concluded that most studies showed higher hemoglobin and ferritin concentrations in obese subjects compared to normal-weight adults (30). Iwasaki’s study gave different results too. They found a positive correlation between SF levels and body fat index in adults (31).

According to our study, women in the highest BF% group demonstrated a significantly higher risk of ID compared to those in the lowest BF% group, which is consistent with Aguree’s finding that obese women have a higher prevalence of ID (6). This also aligns with the outcomes of other researches which showed lower serum iron concentrations in overweight women, but no difference in men (32). There are also studies that yielded different results from our research findings, they claimed that no difference is in serum iron between obese and normal weight controls (33). In our study, we did not find any link between obesity and iron deficiency anemia (IDA). Some obese individuals with hidden or early-stage IDA may not show obvious signs of anemia (19). Depriving developing erythrocytes of iron supply during maturation leads to a reduction in red blood cell production (34). Consequently, this led to reduced erythrocyte synthesis, which may explain the lower MCV values observed in subjects with high BF%.

A major strength of this study is we pioneered the use of BF% instead of BMI to explore the association between obesity and iron deficiency, offering a fresh perspective on this complex relationship. Moreover, the inclusion of a nationally representative sample ensured that our results could be extended to the broader population, enhancing the external validity of our findings. The present study also has limitations. Firstly, cross-sectional design: the use of a cross-sectional design limited our ability to establish causality between obesity and iron deficiency anemia. While we can identify associations, we cannot infer the direction of causation. Secondly, limited male sample size: the insufficient number of male participants in our study hindered the precision and reliability of our extrapolations for this subgroup. Therefore, future prospective study is needed to confirm our findings.

## Data availability statement

Publicly available datasets were analyzed in this study. This data can be found at: https://www.cdc.gov/nchs/nhanes/index.htm (NHANES).

## Ethics statement

This research was exempt from local institutional review board review because of the de-identified data analyzed.

## Author contributions

ZC: Writing – original draft, Data curation. BC: Writing – original draft, Software, Methodology. LL: Writing – original draft, Validation. XT: Writing – review & editing, Supervision. HX: Writing – review & editing, Conceptualization.
